# Structural Characterization and Anti-inflammatory Activity of a Galactorhamnan Polysaccharide From *Citrus medica* L. var. *sarcodactylis*

**DOI:** 10.3389/fnut.2022.916976

**Published:** 2022-06-09

**Authors:** Bi Luo, Jia Lv, Kejie Li, Peiran Liao, Peng Chen

**Affiliations:** ^1^School of Traditional Chinese Medicine, Guangdong Pharmaceutical University, Guangzhou, China; ^2^Key Laboratory of State Administration of Traditional Chinese Medicine for Production & Development of Cantonese Medicinal Materials, Guangzhou, China; ^3^Comprehensive Experimental Station of Guangzhou, Chinese Materia Medica, China Agriculture Research System, Guangzhou, China; ^4^School of Pharmaceutical Sciences, Guangzhou University of Chinese Medicine, Guangzhou, China

**Keywords:** *Citrus medica* L. var. *sarcodactylis*, galactorhamnan polysaccharide, anti-inflammatory activity, antioxidant activity, structure characterization

## Abstract

This study aimed to extract polysaccharides from *Citrus medica* L. var. *sarcodactylis* (finger citron fruits) and analyze their structures and potential bioactivities. A new polysaccharide named K-CMLP was isolated and purified by Diethylaminoethylcellulose (DEAE)-Sepharose Fast Flow and DEAE-52 cellulose column chromatography with an average molecular weight of 3.76 × 10^3^ kDa. Monosaccharide composition analysis revealed that K-CLMP consisted of rhamnose, galactose, and glucose, with a molar ratio of 6.75:5.87:1.00. Co-resolved by methylation and two-dimensional nuclear magnetic resonance (NMR), K-CLMP was alternately connected with 1, 2-Rha and 1, 4-Gal to form the backbone, and a small number of glucose residues was connected to O-4 of rhamnose. The results of *DPPH⋅* and *ABTS^+^⋅* radical scavenging assays indicated that both crude polysaccharide Citrus medica L. var. polysaccharide (CMLP) and K-CLMP exhibited strong free-radical-scavenging properties in a dose-dependent manner. In addition, K-CMLP significantly inhibited the production of pro-inflammatory cytokines (IL-6 and TNF-α) and reactive oxygen species (ROS) in RAW 264.7 cells treated with LPS. These results provide a basis for further use as one of the potential functions of food or natural medicine.

## Introduction

*Citrus medica* L. var. s*arcodactylis* (Noot) Swingle (Finger citron), also known as Buddha’s hand, is a variation of *Citrus medica* L. and belongs to the genus *Citrus*, Rutaceae family. The main producing areas are in the south of China, especially in Guangdong, Fujian, Sichuan, and Zhejiang provinces (1). The fruit of *C. medica* L. var. s*arcodactylis* is widely used as a precious traditional Chinese medicine, perfume raw materials, decorative bonsai, and different types of processed foods such as preserved fruit “laoxianghuang.” As a traditional Chinese medicinal food, it has the functions of depressed liver, harmonizing stomach, and expelling phlegm (2), and it was used as an adjuvant herbal medicine to treat multiple chronic diseases like hypertension, tracheitis, respiratory tract infections, angiocardiopathy, and asthma (3). In recent years, the chemical constituents isolated from the fruits of finger citron include polysaccharides (3, 4), neolignans (2), flavonoids (5), coumarins (6), terpenoids (7), glycosides (2), and other bioactive substances (8), which have revealed a wide variety of biological activities, including antidepressants (9), antibacterial (10), anticancer (11), antiaging (12), antibiofilm (13), antioxidants (14), and anti-inflammatory activities (15).

Especially, as both an edible and a medicinal fruit, it has been confirmed that its many healing properties are attributed to the polysaccharides, one of the major active ingredients of finger citron (16). According to the functional and practical applications of finger citron in folk medicine, the most promising biological properties of these polysaccharides are anti-inflammatory and immunomodulatory activities.

Finger citron, naturally used in China as a medicine with anti-inflammatory and anti-oxidative activity, is widely used because of its edible and medicinal properties. It has been generally confirmed that its health benefits are closely related to the essential oils and flavonoids. Besides essential oils and flavonoids, polysaccharides in finger citron are also important bioactive ingredients. It has been reported to possess antioxidant and immunoregulatory activity (15). As finger citron is usually extracted by hot water when serving as medicine, the water-soluble components, especially polysaccharides, should be responsible for the pharmacological effects (17). However, the structure of polysaccharides is closely related to their pharmacological effects. Few reports are available on the anti-inflammatory activity of polysaccharides derived from finger citron. It is required to reveal which bioactive homogeneous component responsible for the anti-inflammatory and anti-oxidative activity is highly accumulated in finger citron.

This work presents a new galactorhamnan polysaccharide, K-CMLP, isolated and further purified from the crude polysaccharide *via* Diethylaminoethylcellulose (DEAE) sepharose fast flow and DEAE-52 column chromatography. The structure of polysaccharide K-CMLP was elucidated by monosaccharide composition and methylation analysis combined with 1 D and 2D nuclear magnetic resonance (NMR) spectroscopies. We also investigated polysaccharide K-CMLP as an antioxidant against *DPPH⋅* and *ABTS*^+^⋅radicals, the anti-inflammatory activities effect on the production of pro-inflammatory cytokines (TNF-α, IL-6), as well as cell count and reactive oxygen species’ (ROS) scavenging activity in LPS-induced RAW 264.7 cells.

## Materials and Methods

### Plant Materials and Chemicals

The fruit of *Citrus medica* L. var. *sarcodactylis* was harvested in October 2018 from the planting base of Nanling Parmaceutica Co. Ltd., Tongyou village, Pingtang town, Yunfu city (22°46’ N, 111°45’ E), Guangdong, China. The material was identified by associate professor Jinzhu Liu at the School of traditional Chinese medicine, Guangdong Pharmaceutical University, China. Galacturonic acid, D-glucose, trifluoroacetic acid (TFA), T-series Dextran, lipopolysaccharide (LPS), and DEAE-cellulose were purchased from Aladdin Reagent Int. (Shanghai, China). Other chemicals used in this work were analytical grade. Macrophages from the macrophage cell line RAW 264.7 were purchased from Nanjing Kebai Biotechnology Co., Ltd.

### Extraction of Crude Polysaccharide

Fresh fruits were cut into 0.5–1.0 cm palm-shaped slices and dried at 50°C till the moisture content was less than 15%. The dried slices were ground to a powder. The powder (1.00 kg) was depigmented, defatted, and alcohol-soluble ingredients were removed by pretreating with 75% ethanol (1:15, w/v) using the Ultrasound method triple for 1 h. The dried residues were extracted with distilled water (1:10, w/v) at 100°C triple for 2 h and then filtered (18). The combined filtrates were concentrated to dryness under pressure at 55°C. The residue was resolved with deionized water and then was centrifuged. The supernatant was precipitated by adding four times the volume of ethanol and left overnight at 4°C. After centrifugation, the precipitate was re-dissolved in distilled water. The solution was deproteinized by Sevage reagent (chloroform/*n*-butanol 4:1, v/v) according to the published method (19). The deproteinized solution was intensively dialyzed against tap water for 72 h (Mw cut off 1000 Da). Finally, the resulting portion was collected and freeze-dried, and a light brown crude polysaccharide Citrus medica L. var. polysaccharide (CMLP, 132.7 g) of finger citron was obtained. High-performance gel-permeation chromatography (HPGPC) was used to detect the purity of polysaccharide.

### Separation and Purification of Citrus medica L. var. polysaccharide

CMLP was dissolved in deionized water and the mixture was centrifuged. The supernatant was loaded on a DEAE Sepharose Fast Flow column (2.5 cm × 40.0 cm) and eluted with distilled water and different concentrations of gradient NaCl solution (0.1 M, 0.2 M, 0.3 M NaCl) at a constant flow rate consecutively. Each fraction was detected by using phenol–sulfuric acid method and then the peak was merged independently. The water elute was further purified by DEAE-52 cellulose column (5 × 50 cm, OH^–1^ form) and eluted with distilled water at a flow rate of 0.8 mL/min, and the main polysaccharides fraction was collected, dialyzed, and lyophilized. Consequently, a white fluffy pure polysaccharide namely K-CMLP (7.71 g) was obtained. The K-CMLP solution was filtered through 0.22-μm membrane and analyzed by HPGPC.

### Molecular Weight Analysis of K-CMLP

The molecular weight of K-CMLP was determined by HPGPC with three columns (Waters Ultra hydrogel 250, 1000, and 2000; 30 cm × 7.8 mm; 6 μm particles) in series. T-series Dextran standards with defined molecular masses were used to calibrate the HPGPC system (20).

### Analysis of Chemical Compositions

Total carbohydrate content was determined by the phenol–sulfuric acid method with D-glucose as the standard (21). Uronic acid content was determined according to the *m*-hydroxydiphenyl-sulfuric acid method and galacturonic acid as the standard (22). Protein content was estimated by Bradford’s with bovine serum albumin (BSA) as the standard (23). The monosaccharide compositions of K-CMLP were detected by high-performance liquid chromatography (HPLC), with 1-phenyl-3-methyl-5-pyrazolone (PMP) pre-column derivatization. Briefly, polysaccharide (10.0 mg) samples were hydrolyzed with 3 ml of 4 M TFA at 110°C for 4 h. Then, the residue was washed with methanol and lyophilized several times until TFA was removed completely after the solution was concentrated under vacuum. The sugar residues after hydrolysis were dissolved in distilled water, and PMP methanol solution and NaOH solution were added to the hydrolyzed samples for derivatization. The mixture was neutralized with HCl. Subsequently, chloroform was added and extracted in triplicate and the organic phase was discarded. Finally, the solution was analyzed using an HPLC system equipped with a COSMOSIL 5C18-PAQ column (4.6 × 150 mm, 5 μm), which was eluted with the mobile phase of a 0.05 M KH_2_PO_4_ (pH = 6.9) and acetonitrile in the volume ratio of 80:20 at 1.0 mL min^–1^.

### Methylation Analysis

To define the glycosyl linkages, K-CMLP was methylated according to the method of Hakomori (24) with slight modifications. KMCP (10.0 mg) was added to a suspension of NaH (1.2 equiv) in dry DMSO (5.0 mL/mmol) stirred under a nitrogen atmosphere, and the reaction mixture was stirred at room temperature overnight. Then 1.5 mL of methyl iodide was slowly added dropwise into the mixed solution in an ice bath with the ultrasonic method. The disappearance of the OH band in the FT-IR spectrum (3000–3400 cm^–1^) was used to confirm complete methylation. Fully methylated polysaccharide was dissolved and hydrolyzed in 5 mL 2 M TFA at 110°C for 2 h. After cooling at r.t., methanol was added and evaporated to dryness to remove extra TFA. The residue was dissolved in 3 mL distilled water, and 30 mg NaBH_4_ was added for reduction, 25%HOAc was used to neutralize until gas formation ceased. Subsequently, the spin-dried sample was acetylated with acetic anhydride at 110°C for 1 h. After the solution was extracted with the chloroform–water system three times and the chloromethane phases were collected, the methylated alditol acetate was obtained and detected by gas chromatography-mass spectrometry (GC-MS).

### Nuclear Magnetic Resonance Analysis

The nuclear magnetic resonance of polysaccharide K-CMLP was performed with the method reported in Refs. (25, 26). Polysaccharide K-CMLP (30.0 mg) was dissolved in D_2_O and freeze-dried several times to exchange H protons into deuterium completely. Subsequently, polysaccharide K-CMLP was dissolved in D_2_O overnight before NMR analysis with TSP as the calibration standard. ^1^H NMR, ^13^C NMR, ^1^H-^1^H correlation spectroscopy (COSY), hetero-nuclear singular quantum correlation (HSQC), hetero-nuclear multiple bond correlation (HMBC) spectra were recorded with a Bruker Avance-600 NMR spectrometer (Bruker Instrumental Inc., Bremen, Germany), with a probe temperature of 25°C. The acquisition times were set to 64 times for ^1^H NMR spectra, 5,120 times for ^13^C NMR spectra, 64 times for ^1^H-^1^H COSY, 64 times for HSQC, and 128 times for HMBCspectra. The ^1^H was recorded in the F2 channel with a 10.0 ppm spectrum width and ^13^C was tested in the F1 channel with a 180.0 ppm spectrum width. The spectra were processed using MestReNova v14.0.0-23239 (Mestrelab Research, Santiago de Compostela, Spain) software. For correct peak integration, the spectra were previously baseline-corrected with the default option.

### Antioxidant Activity of Citrus medica L. var. polysaccharide and K-CMLP

#### *DPPH*⋅Free Radical Scavenger

*DPPH*⋅radical-scavenging activity was evaluated with a method reported in Huang and Huang (27). In brief, 20 μL of CMLP and K-CMLP solutions (0–10.0 mg/mL) were mixed with 180 μL of *DPPH⋅* of ethanol solution (0.1 mM). Ascorbic acid (Vc) was used as the positive control. The mixtures have been shaken immediately and incubated in darkness for 30 min, and a value of A_517_ nm was detected using a microplate reader against a control containing 20 μL distilled water and 180 μL *DPPH⋅* solution.

#### *ABTS*+⋅ Free Radical Scavenger

Assessment of *ABTS*^+^⋅ radical-scavenging activity was done according to a previously published method (28). A total of 7 mmol/L *ABTS*^+^⋅and 2.45 mmol/L K_2_S_2_O_8_ at the volume ratio of 1:1 was mixed and incubated in a dark place for 16 h at room temperature. The mixture was diluted with PBS at pH 7.4 to give an absorbance of 0.7 ± 0.02 at 734 nm. Then *ABTS*^+^⋅solution (160 μL) was added to CMLP or K-CMLP solution at different concentrations. The reaction mixture was kept at room temperature for 6 min before measuring the absorbance at 734 nm.

### Anti-inflammatory Activity of K-CMLP

#### Measurement of Cytotoxicity and Cytokines

RAW 264.7 macrophages were cultured in DMEM supplemented with 10% FBS and 100 U/mL of penicillin–streptomycin (double antibody) at 37°C in an atmosphere of 5% CO_2_. RAW 264.7 macrophages (1 × 10^6^ cells/well) were pretreated with K-CMLP whose final concentration was 50 μg/mL and stimulated with or without LPS (0.1 μg/mL) for 24 h at 37°C. The cell cytotoxicity of K-CMLP was tested by the MTT test. Levels of TNF-α and IL-6 were quantified using ELISA kits (Solarbio, Beijing, China) according to the manufacturer’s instructions and a standard curve to calculate it. Briefly, samples were added in a captured antibody-coated 96-well plate for 60 min at 37°C. After washing, the detected antibody was incubated for 30 min at 37°C and HRP was reacted for 30 min at 37°C. The wells were filled with 100 μL substrate solution for 15 min in the dark and then a stop solution was added to terminate the reaction. The absorbance was measured at 450 nm using a microplate reader.

#### Analysis of Intracellular Reactive Oxygen Species Production

The level of cellular ROS formation was assessed with the ROS assay kit. After treatment, the RAW 264.7 cells were collected and washed with PBS. 2′,7′-Dichlorodihydrofluorescein diacetate (DCFH-DA) is a cell-permeable probe (Ex/Em = 488/530 nm) for detecting intracellular ROS. The cell culture medium was removed and the cells were incubated with 10 μM DCFH-DA at 37°C for 20 min. After washing three times with serum-free cell culture medium, fluorescence emission (525 nm) was measured using a 488 nm laser and 530/30 filter on a BD LSRFortessa flow cytometer.

### Statistical Analysis

Data for quantification were acquired from individual experiments repeated at least three times and were expressed as the means ± SD. Statistical significance was calculated by GraphPad Prism 7.00 software (GraphPad Software, Inc., San Diego, CA, United States) with unpaired two-tailed t-tests and accepted by *p* < 0.05 (*), *p* < 0.01 (^**^), *p* < 0.001 (^***^), *p* < 0.0001 (^****^). The IC_50_ was calculated using the GraphPad Prism 7.00 software according to the inhibition rates or reduction rates (y) plotted against the sample concentrations (x).

## Results and Discussion

### Characterization of Citrus medica L. var. polysaccharide and K-CMLP

**FIGURE 1:**
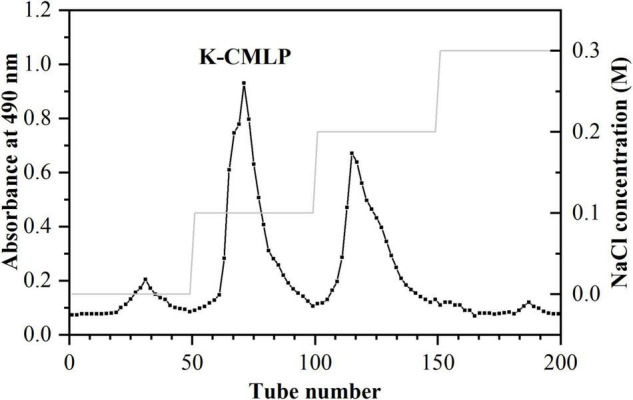
DEAE cellulose-52 fractionation profile of polysaccharide K-CMLP.

**FIGURE 2:**
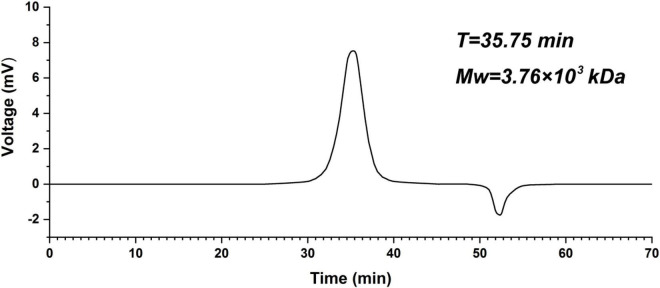
High-performance gel-permeation chromatography chromatogram of polysaccharide K-CMLP.

The crude polysaccharide was extracted from the fruit of bergamot with a yield of 13.27%. After alcohol precipitation, dialysis, DEAE Sepharose Fast Flow column, and DEAE-52 column chromatography, the elution curve is shown in Figure 1, a homogeneous polysaccharide of bergamot was obtained, named K-CMLP, and the yield was 5.81%. Measured by HPGPC, the chromatographic peak of K-CMLP was a single symmetrical peak, indicating that the purity of K-CMLP was very high (Figure 2). According to the dextran standard, the molecular weight-retention time standard curve was log(Mw) = –0.1841T + 12.1568, *R*^2^ = 0.9843, and the calculated molecular weight of K-CMLP was 3.76 × 10^3^ kDa.

As shown in Table 1, the results of the chemical composition of polysaccharide show that K-CMLP was mainly composed of neutral sugars, and the content of protein and uronic acid was less than 2%. The monosaccharide composition of K-CMLP was measured using pre-column PMP derivatization by HPLC. The results showed that K-CMLP was identified as an galactorhamnan which was mainly composed of rhamnose and galactose with a relative molar ratio of 49.88 and 43.38% and also contained a small amount of glucose in a molar ratio of 7.39%.

**TABLE 1:** Composition analysis of bergamot polysaccharide.

Item	CMLP	K-CMLP
Carbohydrate (%)	96.35 ± 4.23	98.36 ± 2.35
Protein (%)	–	0.14 ± 0.06
Uronic acid (%)	3.03 ± 0.35	1.57 ± 0.45
Monosaccharide composition (%)		
Glc	15.64	7.39
Rha	27.54	43.38
Ara	8.3	–
Gal	39.73	49.88
Gal A	8.79	–

### Monosaccharide Composition of K-CMLP

**FIGURE 3:**
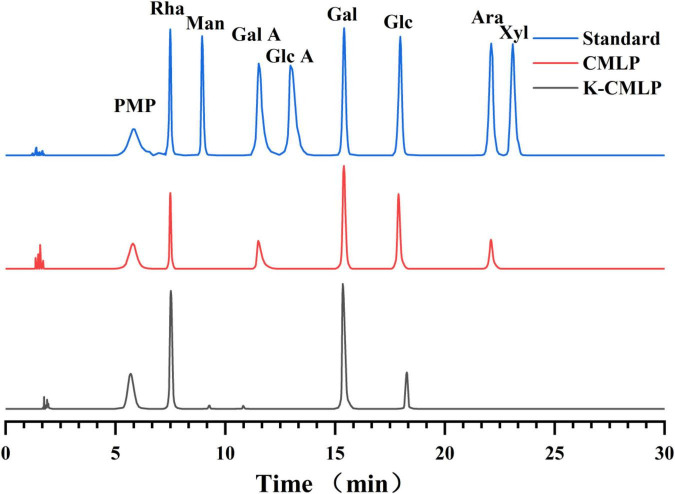
High-performance liquid chromatography pre-column PMP derivative chromatogram of mixed monosaccharide standard, CMLP, and K-CMLP.

The monosaccharide composition of K-CMLP was determined by HPLC-PAD (Figure 3). The presence of rhamnose, galactose, and glucose in K-CMLP were at the molar ratio of 6.75:5.87:1.00, indicating that K-CMLP was a type of heteropolysaccharide.

### Methylation and Gas Chromatography-Mass Spectrometry Analysis of K-CMLP

**FIGURE 4:**
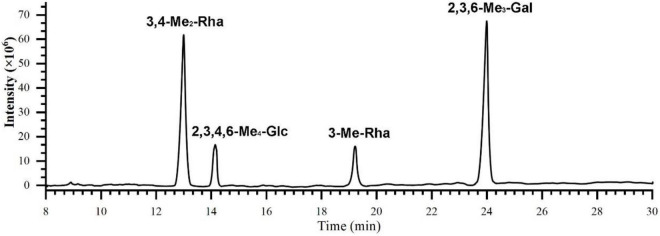
The total ion chromatogram of K-CMLP by GC-MS.

Methylation analysis is an indispensable experimental method to study the types of glycosidic bonds in polysaccharides. In this study, polysaccharide K-CMLP structural analysis was performed using methylation analysis in combination with one-dimensional and two-dimensional nuclear magnetic resonance spectroscopy. After methylation, the fully methylated K-CMLP was hydrolyzed with acid, converted into alditol acetates, and analyzed by GC-MS. As shown in Table 2 and Figure 4, the presence of four major alditol acetate compounds, 3, 4-Me_2_-Rha, 3-Me-Rha, 2, 3, 6 -Me_3_-Gal, and 2, 3, 4, 6-Me_4_-Glc, which indicate the presence of 1, 2-Rha, 1, 2,4-Rha, 1, 4-Gal, and T-Glc in a ratio of 3.9:1.0:4.8:1.1. The contents of 1, 2,4-Rha and T-Glc were basically the same, indicating that the methylation results were reliable.

**TABLE 2:** Methylation analysis of restored K-CMLP.

Methylation sugar	Ratio	Linkage type	Mass fragments (m/z)
3, 4-Me_2_-Rha	3.9	1, 2-Rha	43,57,59,72,88,89,100,115,130,131,160,174,190
3-Me-Rha	1.0	1, 2,4-Rha	43,59,69,74,88,101,130,143,160,171,190,203
2, 3, 6-Me_3_-Gal	4.8	1, 4-Gal	43,59,71,87,99,102,113,118,129,131,142,162,173,188,203,233
2, 3, 4, 6-Me_4_-Glc	1.1	T-Glc	43,59,71,75,87,88,101,102,113,118,129,145,161,162,175,205

### Nuclear Magnetic Resonance Spectroscopy Analysis

**FIGURE 5:**
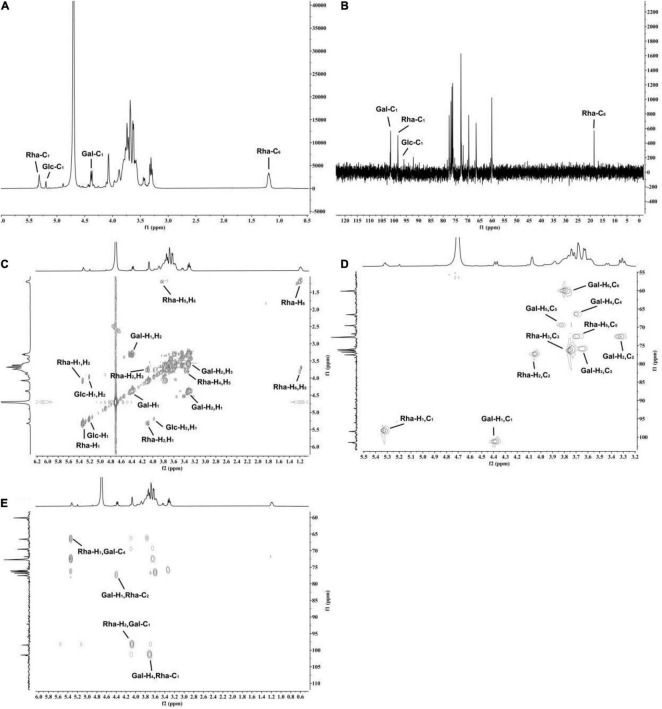
The NMR spectroscopy of polysaccharide K-CMLP. ^1^H NMR **(A)**, ^13^C NMR **(B)**, ^1^H- ^1^H COSY **(C)**, ^1^H-^13^C HSQC **(D)**, and ^1^H-^13^C HMBC **(E)**.

**FIGURE 6:**
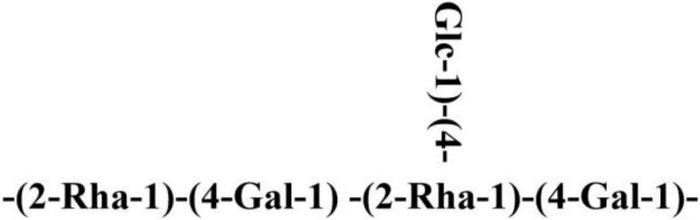
Schematic diagram of polysaccharide K-CMLP.

According to the ^1^H NMR and ^13^C NMR nuclear magnetic resonance spectra (Figure 5A), it was shown that K-CMLP had 3 signal peaks in the anomeric hydrogen region (4.3–5.5 ppm), and the chemical shifts were δ4.38, δ5.32, and δ5.21 ppm, respectively. Combining with the results of monosaccharide composition (Figure 5B), it was concluded that these three anomeric hydrogens belong to β-Gal*p*, α-Rha*p*, and α-Glc*p*, respectively, and the chemical shifts of β-Gal*p*, α-Rha*p*, and α-Glc*p* of ^13^C NMR were δ101.1, δ98.5, and δ96.1 ppm, respectively. The absorption peak at 18.41 ppm was the typical methyl signal peak of rhamnose. The chemical shifts of carbon and hydrogen on K-CMLP were assigned by ^1^H-^1^H COSY (Figure 5C) and HSQC (Figure 5D), and the results are summarized in Table 3. In the HMBC spectrum (Figure 5E), the (Gal-H4, Rha-C1) and (Rha-H1, Gal-C4) cross-peaks indicated that the rhamnose residue was connected to O-4 of galactose. The cross-peaks (Gal-H1, Rha-C2) and (Rha-H2, Gal-C1) indicated that the galactose residue was attached to O-2 of rhamnose. The structure of polysaccharides was very complex, and only the repetitive units in polysaccharides could be analyzed by methylation analysis and NMR analysis. However, more accurate structural information on polysaccharides needs to be combined with a lot of structural verification, such as partial acid hydrolysis, Smith degradation and so on. In summary, the repeating units of K-CMLP main chain were 1, 2-Rha and 1, 4-Gal alternately connected, and a small amount of glucose residues were connected to O-4 of rhamnose (Figure 6). The polysaccharide K-CMLP contains a large number of hydroxyl groups, forming intramolecular and intermolecular hydrogen bonds in an aqueous solution (29), so that they have strong water retention because the complex three-stage network structure formed by its intermolecular action has an aqueous solution with remarkable viscoelasticity (30, 31). It has been reported that enhancing the viscosity of the digesta could help some physiological responses, including constipation relief and blood glucose control (32), which suggested that the polysaccharide K-CMLP might help control blood glucose and cholesterol levels.

**TABLE 3:** Chemical shift assignment of K-CMLP.

Code	^1^H/^13^C NMR δ [ppm]					
	
	1	2	3	4	5	6
Gal	4.38	3.30	3.58	3.62	3.75	3.73
	101.1	72.5	75.8	66.4	69.3	60.1
Rha	5.32	4.07	3.68	–	3.63	1.18
	98.5	77.6	76.5	–	72.4	18.4
Glc	5.21	3.97	–	–	–	–
	96.1	–	–	–	–	–

*(–) Polysaccharide resonances were not assigned due to low intensity and overlap with other resonances.*

### Antioxidant Activity of Citrus medica L. var. polysaccharide and K-CMLP

**FIGURE 7:**
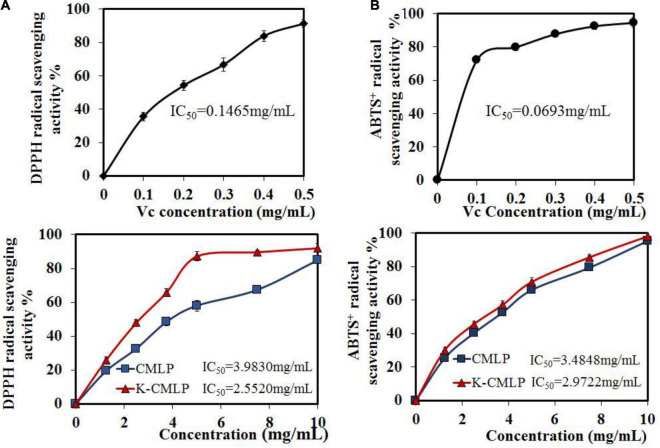
Antioxidant activities of CMLP and K-CMLP: *DPPH⋅* radical-scavenging activity **(A)**; *ABTS*^+^⋅ radical-scavenging activity **(B)**. Values are the mean ± SD of three replicates.

The *DPPH⋅* and *ABTS^+^⋅* tests were widely used to evaluate the ability of compounds to scavenge free radicals *in vitro* (33). As shown in Figure 7A, CMLP and K-CMLP were able to scavenge *DPPH⋅* radicals to different degrees in a dose-dependent manner when the concentration ranged from 0 to 10.0 mg/mL. The higher *DPPH⋅* scavenging activity was displayed by the homogeneous polysaccharide K-CMLP with IC_50_ = 2.5520 mg/mL. Obtained results showed that K-CMLP is the main component in the crude polysaccharide which has antioxidant activity. In the case of *ABTS*^+^⋅ scavenging activity, the various samples showed the same trend, and K-CMLP was the most effective compared with CMLP (IC_50_ = 2.9722 mg/mL) (Figure 7B). These results revealed that K-CMLP contains many hydroxyl groups, with high hydrogen-donating capacity.

### Anti-inflammatory Activity of K-CMLP

**FIGURE 8:**
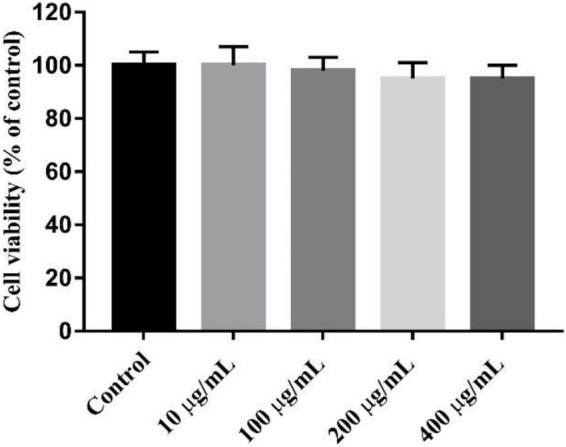
Cytotoxicity of K-CMLP in RAW 264.7 macrophages at the different concentration (10, 100, 200, 400 μg/mL).

**FIGURE 9:**
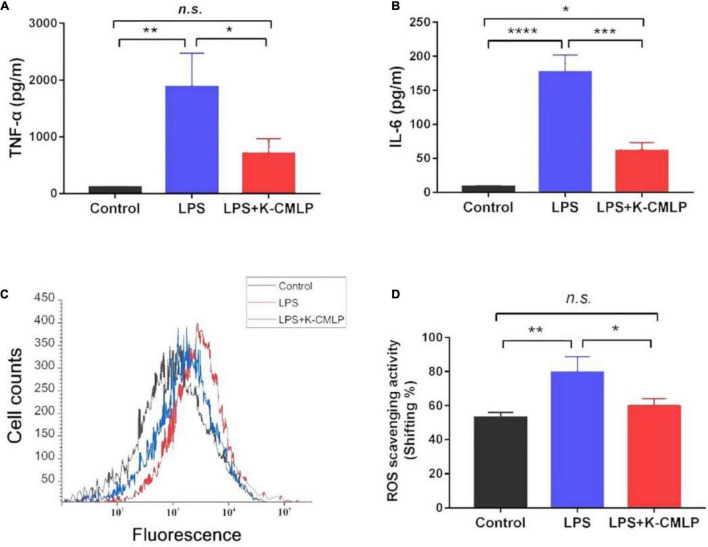
Anti-inflammation effect of K-CMLP on production of pro-inflammatory cytokines [TNF-α **(A)**, IL-6 **(B)**], cell counts **(C)** and reactive oxygen species (ROS) scavenging activity **(D)** in LPS induced RAW 264.7 cells model. Values represent the mean ± SE (bars) from eight independent samples (*n* = 3). * Means *P* < 0.05, ** means *P* < 0.05, ***P < 0.0005, and ****P < 0.0001 compared with the only LPS treated group for incubating 24 h.

The MTT test showed that K-CMLP had no cytotoxicity to RAW 264.7 macrophages at different concentrations (10, 100, 200, and 400 μg/mL), and cell viability was more than 95% (Figure 8). Macrophages, important components in the human immune defense system, respond actively to inflammation by releasing pro-inflammatory cytokines, such as TNF-α, IL-1β, and IL-6; high levels of these cytokines can cause systemic complications (34, 35). Lipopolysaccharide (LPS) was an outer membrane component of Gram-negative bacteria that can cause severe inflammation by triggering the production of various proinflammatory cytokines. When LPS was added to the cells, TNF-α and IL-6 increased significantly (36). However, the production of LPS-induced TNF-α (*P* < 0.05 Figure 9A) and IL-6 (*P* < 0.0005, Figure 9B) was significantly inhibited by the polysaccharide K-CMLP. Both systemic and local inflammation may foster an oxidative injury with the release of ROS (37). To investigate whether the anti-inflammatory effect of polysaccharide K-CMLP was related to its antioxidant activity, the production of ROS detected by the fluorescent probe DCFH-DA was evaluated. As shown in Figures 9C,D, LPS treatment significantly increased the ROS production of RAW 264.7 cells. However, when treated with the polysaccharide K-CMLP, the production of ROS was significantly inhibited. These data support the hypothesis that the anti-inflammatory effect of polysaccharide K-CMLP may be related to its antioxidant capacity.

## Conclusion

In this study, a novel high molecular polysaccharide K-CMLP was purified from finger citron. K-CMLP is a new type of galactorhamnan with a molecular weight of 3.76 × 10^3^kDa. The main linkage types of K-CMLP were 1,2,4-Rha-linked-1, 4-Gal and were substituted by β-D-Gal*p* units at 4-OH of rhamnose. Up to now, the proportion of rhamnose in the reported polysaccharides isolated from finger citron is relatively less than arabinose, galactose, glucose, and xylose. Overall, K-CMLP was a new type of galactorhamnan. Both CMLP and K-CMLP exhibited potential antioxidant activities *in vitro*. Our findings suggest that K-CMLP is able to inhibit the production of pro-inflammatory cytokines (TNF-α, IL-6), as well as ROS in LPS-stimulated RAW 264.7 macrophages. In conclusion, this is the first report describing a novel galactorhamnan polysaccharide present in finger citron fruit, and the bioactivity data suggest that K-CMLP could be used as a function food for health.

## Data Availability Statement

The original contributions presented in this study are included in the article/supplementary material, further inquiries can be directed to the corresponding authors.

## Author Contributions

BL and PC contributed to the conception, design, and funding of the study. BL completed the purification of the polysaccharide and identified its structure. JL completed the antioxidant assay of polysaccharides. KL completed the anti-inflammatory assay of polysaccharides. PL and PC completed the writing and revision of the manuscript. All authors contributed to the article and approved the submitted version.

## Conflict of Interest

The authors declare that the research was conducted in the absence of any commercial or financial relationships that could be construed as a potential conflict of interest.

## Publisher’s Note

All claims expressed in this article are solely those of the authors and do not necessarily represent those of their affiliated organizations, or those of the publisher, the editors and the reviewers. Any product that may be evaluated in this article, or claim that may be made by its manufacturer, is not guaranteed or endorsed by the publisher.
